# Factors Associated with Stage at Diagnosis in Pancreatic Cancer: Implications for Precision Screening and Early Detection

**DOI:** 10.3390/biomedicines14050992

**Published:** 2026-04-27

**Authors:** Elen Deng, Manvita Mareboina, Ilias Georgakopoulos-Soares, Nelson S. Yee

**Affiliations:** 1 Department of Molecular and Precision Medicine, Institute for Personalized Medicine, The Pennsylvania State University College of Medicine, Hershey, PA 17033, USA; edeng@pennstatehealth.psu.edu (E.D.); mmareboina@pennstatehealth.psu.edu (M.M.); 2 Cancer Control Program, Penn State Cancer Institute, Hershey, PA 17033, USA; 3 Department of Medicine, Division of Hematology-Oncology, Penn State Health Milton S. Hershey Medical Center, Hershey, PA 17033, USA; 4 Experimental Therapeutics Program, Penn State Cancer Institute, Hershey, PA 17033, USA

**Keywords:** pancreatic cancer, stage at diagnosis, social determinants of health, disparities early detection, screening, biomarkers, extracellular vesicles, machine learning, electronic health records

## Abstract

Pancreatic cancer is a leading cause of cancer-related mortality in the United States and worldwide. Most patients are diagnosed with pancreatic cancer at advanced stages, when curative therapy is no longer possible. The stage of pancreatic cancer at diagnosis critically impacts the treatment options and thus the clinical outcomes. Currently, there is no established screening program or tests for its early detection. Studying and understanding how those factors influence the stage of pancreatic cancer at diagnosis helps identify barriers and develop screening strategies. Tumoral and demographic factors, as well as social determinants of health, tend to be associated with localized vs. advanced stage of pancreatic cancer at diagnosis. Socioeconomic factors have been shown to be important mediators of racial disparities in stage at diagnosis as well as germline genetic testing. Recently, screening initiatives, blood-based molecular biomarker tests for early detection of pancreatic cancer, and machine learning-based models for risk prediction and imaging diagnostics have been developed. By determining and understanding the factors associated with the stage at diagnosis, risk-stratified screening can be feasible by combining demographics, genetics, comorbidities, lifestyle, and social determinants. Moreover, regulatory policies that address the social determinants of health can guide the development of screening strategies to allocate resources for equitable access to healthcare and to reduce disparities in patients with pancreatic cancer.

## 1. Introduction

Pancreatic cancer (PC) has remained a medical and public health challenge worldwide [1]. It is the third leading cause of cancer-related death per new cases in the United States and worldwide after lung and colorectal cancer [2,3]. The incidence of PC has been increasing in recent decades, especially among young adults (aged 25–49 years) [4,5]. It represents approximately 3% of all cancer cases and 7% of cancer deaths in the United States, with a lowest five-year survival rate of 13% [1,2,5]. When diagnosed at early stages, PC is associated with increased 5-year survival rates—localized 44%, regional 16%, and distant 3% [2]. Pancreatic tumors without involvement of the regional vasculatures are considered resectable and associated with relatively high survival rates. However, fewer than 20% of patients are diagnosed early enough for surgery with curative intent [6]. For the majority of patients with advanced or metastatic disease, palliative systemic or targeted therapy and radiation therapy with limited efficacy are the only treatment options.

While developing preventive interventions and curative treatments are effective approaches to reduce the mortality rate of PC, screening for early detection of this disease is expected to make a significant impact on its clinical outcomes through timely, effective treatment. Due to a lack of screening tests for early detection of PC and effective treatment of advanced disease, defining and understanding the factors associated with the stage of PC at diagnosis is crucially important [7]. Currently, there is no test proven to be effective for massive screening of PC in an asymptomatic population, or even for individuals at high risk for developing PC. Considering the relatively low incidence of PC in the general population, risk-stratified screening for early detection of this disease will be urgently needed. Recently, multi-institutional consortia have been developed as screening initiatives for PC. Moreover, blood-based tests for early detection of multiple cancers, including PC, have emerged, and machine learning-based models for risk prediction and imaging diagnostics have been developed. However, real-world evidence for the clinical benefit of these multi-cancer early detection tests and machine learning-based algorithms remains to be demonstrated.

In this study, the factors associated with the stage of PC at diagnosis, as well as the known risk factors of developing PC, are evaluated in detail. The screening initiatives for PC and the emerging multi-cancer detection tests for early detection of PC are critically examined. An overview of the application of machine learning-based analysis of electronic health records for risk prediction and improvement of diagnostic accuracy of imaging studies is provided. Understanding the factors associated with the stage of PC at diagnosis and combining the various screening tests and modalities for its early detection will hopefully help develop equitable and targeted interventions for improving early detection, diagnosis, and treatment, and thus clinical outcomes for patients with PC at all stages.

## 2. Methodology

This article presents an updated narrative review of factors associated with the stage of PC at diagnosis, screening initiatives and tests, as well as the application of machine learning for early detection of PC. All authors conducted the search.

Published studies between the years 2000 and 2025 about factors associated with the stage of pancreatic cancer at diagnosis, including original and review studies indexed in PubMed, EMBASE, Scopus, Cochrane Library, and SEER-based literature via Google Scholar, were evaluated. The keywords used to search are “pancreatic cancer” AND “risk factors”; “pancreatic cancer” AND “factors” AND “stage at diagnosis”; “pancreatic cancer” AND “social determinants of health” AND “stage at diagnosis”. Of the 265 records screened for direct relevance and excluded after review of title/abstract, 31 full-text articles are eligible and cited.

Inclusion criteria include adults ≥18-year-old diagnosed with pancreatic (ductal) adenocarcinoma, newly diagnosed and treatment-naïve patients, and studies reporting stage at diagnosis (localized, locally advanced, metastatic, or early vs. late stage), studies evaluating factors associated with stage at diagnosis, observational/interventional/review studies, within the United States, English language, and studies reporting stage distribution by exposure variable. Exclusion criteria include non-adenocarcinoma histology, no stage at diagnosis reported, only survival or treatment outcomes, recurrent or previously treated disease, and case reports/editorials/commentaries/conference abstracts.

For screening initiatives and tests as well as application of machine learning for early detection of PC the search terms used to search are “pancreatic cancer” AND “screening”; “pancreatic cancer” AND “early detection”; “pancreatic cancer” AND “artificial intelligence” AND “early detection”; “pancreatic cancer” AND “machine learning” AND “early detection”. The articles from the search are cited in the Reference section of this manuscript.

## 3. Factors Associated with Stage of Pancreatic Cancer at Diagnosis

PC is often diagnosed at an advanced stage, largely because early disease is asymptomatic and current screening is limited. The stage at diagnosis is influenced by a combination of tumor-related, patient-level, social determinants of health, and healthcare system factors. Tumor-related factors depend on the aggressiveness of biology, location, and molecular features. Patient-level factors include sex, race, and ethnicity, as well as hereditary, medical, and lifestyle factors. Social determinants of health include socioeconomic status, insurance status, and residential location. Healthcare systemic factors primarily relate to a lack of an effective population-wide screening strategy. How those associated factors interact with one another and the mediating mechanisms are not well understood. Interventions and solution-oriented approaches to address those factors, particularly the social determinants of health through health policies, will not only improve screening and early detection of PC but also reduce disparities in the care of PC.

## 4. Tumor-Related Factors and Stage of Pancreatic Cancer at Diagnosis

Many of the symptoms of PC are non-specific, and it is often asymptomatic in its earliest stages. The symptoms of patients with PC can be related to the location and size of the tumor, and tumor-associated symptoms typically become noticeable as the tumor grows and spreads to regional and distant organs and tissues [8]. Symptoms such as fatigue, anorexia, weight loss, abdominal pain, or diarrhea are often overlooked or attributed to other conditions [9]. Tumors in the head of the pancreas often cause symptoms such as jaundice, pruritus, dark-colored urine, and acholic stool, which may lead to prompt medical attention and diagnosis. Tumors in the body or tail of the pancreas may not cause any specific symptoms until late in the disease course, when tumor metastasis has usually occurred, as reviewed [10]. Thus, education of the general population about the signs and symptoms of PC may help improve its diagnosis at an earlier stage.

Educational strategies may include (i) public campaigns to emphasize persistent, unexplained symptoms rather than rare or non-specific complaints; (ii) high-impact visual public campaigns through social media, pharmacy and primary care waiting room posters, community centers, workplaces, and faith organizations; (iii) targeting high-risk groups such as age >50, new onset-diabetes, smokers, chronic pancreatitis, family history or hereditary cancer syndromes, obesity or long-standing diabetes by reaching them in endocrinology and diabetes clinics, smoking cessation programs, gastrointestinal and primary care clinics, and employee wellness programs; (iv) educating primary care providers alongside the public; (v) partnering with organizations such as Pancreatic Cancer Action Network, American Cancer Society, World Pancreatic Cancer Coalition, National Pancreas Foundation, and providing ready-to-use toolkits, media templates, and community outreach infrastructure; (vi) leveraging healthcare system through electronic health record patient portal messages, automated pharmacy alerts, and education of laboratory-result.

In addition, advances in understanding the molecular alterations in PC and distinct molecular subtypes of PC have provided new insights into tumor biology, including growth rate and kinetics of tumor progression. These biological features determine tumor behavior and potentially influence the stage of PC at diagnosis. In the canonical progression model of PC, activation of oncogenic driver KRAS is involved in tumor initiation, loss of tumor suppressor CDKN2A/p16 in proliferation, the tumor suppressor *TP53* mutation in genomic instability, and loss of tumor suppressor SMAD4 in metastatic dissemination [11]. In particular, activating mutations in KRAS correlate with tumor growth and aggressiveness and, when cooperating with other mutations, drive metastatic dissemination. Loss-of-function mutation in p53 is strongly associated with genomic instability and rapid tumor progression, and epithelial–mesenchymal transition; co-mutation of KRAS and p53 produces a highly aggressive, metastatic phenotype. Loss of CDKN2A/p16 correlates with rapid proliferation and early progression from precursor lesions to invasive cancer; it enables expansion of clones that acquire additional metastatic traits. Moreover, subtypes of PDAC have been defined based on integrated genomic/transcriptomic analyses. The basal-like/squamous subtype is enriched for mutations in p53 and activation of MYC, and it is characterized by poor differentiation, high potential for metastasis, and chemoresistance. The classical subtype is associated with epithelial differentiation, and it often retains partial SMAD signaling. How those genomic mutations in *KRAS*, *CDKN2A*, *TP53*, and *SMAD4*, as well as the tumor subtypes of PC, are associated with the stage of PC at diagnosis remains to be determined. Conceivably, integrating the genomics and tumor subtypes with clinical data may help improve the prediction of early metastasis and stage at diagnosis.

## 5. Sex/Race/Ethnicity and Stage of Pancreatic Cancer at Diagnosis

In the United States, the incidence of PC is generally higher in men than in women [1,12,13,14,15]. The incidence of PC is 50–90% higher in Black individuals compared to Non-Hispanic Whites and Hispanic/Latinos [12]. Among each ethnic group, including White, Black, American Indian/Alaska Native, Asian/Pacific Islander, and Hispanic, the incidence of PC is invariably higher in men than women [14,15].

Epidemiological studies indicate that the stages of PC at diagnosis vary with sex, race, and ethnicity. In the study by Saif et al. (2005), no significant difference in the incidence of PC diagnosed at all stages was found between men and women [16]. Early onset pancreatic cancer (EOPC) diagnosed in patients under 50 years old was found to be slightly more common in men (54.2%) as compared with women 45.8% [17].

However, Gallegos et al. (2023) demonstrated that women were more likely to be diagnosed at localized stages (I and II) than men (odds ratio 0.857; 95% CI = 0.839–0.875; *p* < 0.001); men were more frequently diagnosed with advanced stages (III and IV) of PC as compared to women (52.8% vs. 47.9%; *p* < 0.001) [18]. In addition, a number of studies demonstrated racial and ethnic disparities in the stage of PC at diagnosis, as shown in Table 1.

Thus, the stage of pancreatic cancer at diagnosis varies with sex, race, and ethnicity, with Black men tending to present with PC at an advanced stage than Whites and women. The discrepancies in findings across those studies may be related to the study populations and geographical locations. However, various factors may account for the sexual and racial/ethnic disparities in the stage of PC at diagnosis, such as sex hormones, germline mutations, somatic mutations, patterns of DNA methylation, and social determinants. For instance, the study by Saif et al. (2005) [16] using a database of a single institution showed no significant difference in the distribution of tumor stage between Whites and Blacks. Gallegos et al. (2023) [18] found that other races have 15% higher odds of an early-stage disease compared to Whites, and this finding is not truly a population-based analysis. In the study by Schiefelbein et al. (2022) [23], a greater proportion of non-Hispanic Blacks were diagnosed with localized disease than non-Hispanic Whites, whereas non-Hispanic Blacks were diagnosed with distant disease at similar proportions. The apparent discrepancies among these studies could be attributed to the multi-level, interacting factors that influence one another. However, the mechanisms that underlie the sexual and racial/ethnic disparities in the stage of PC at diagnosis may be determined by (i) examining the contribution of behavioral factors and comorbidities such as smoking, diabetes, obesity, pancreatitis; (ii) healthcare access including health insurance, primary care visits, residential locations, income, education levels, language, geographic access to imaging evaluation; (iii) time from symptom to imaging and time from imaging to diagnosis; (iv) biological factors including tumor location, histological and molecular features, prevalence of new-onset diabetes and chronic pancreatitis, utilization patterns of healthcare including preventive care, continuity of care, number of clinical encounters prior to diagnosis, and emergency department-based diagnosis. Studying the mechanisms underlying the sexual and racial/ethnic disparities in the stage of PC at diagnosis, as well as the interactions among the mechanisms, will enable the generation of hypotheses to be tested.

## 6. Hereditary, Medical, and Lifestyle Factors and Stage of Pancreatic Cancer at Diagnosis

**Figure 1:**
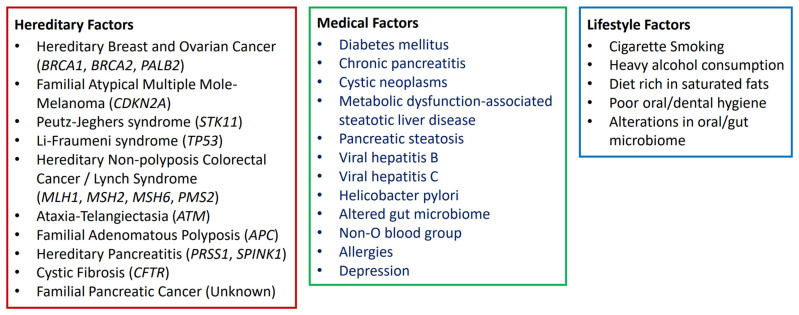
Non-modifiable and modifiable risk factors of pancreatic cancer.

Certain known risk factors for PC have been identified, and they are associated with genetic abnormalities, lifestyle, and morbidities (Figure 1). Non-modifiable risk factors include blood groups, allergies, and hereditary syndromes that involve specific genetic mutations, as reviewed [24,25,26,27,28]. Some of the risk factors are potentially modifiable, and they are related to lifestyle and behaviors as well as morbidities that may be manageable.

How these risk factors are associated with the stage of PC at diagnosis is unclear. However, individuals with familial pancreatic cancer or genetic syndromes are advised to undergo screening or surveillance for PC or other cancers at an early age and increased frequency [29]. This may lead to early diagnosis of PC as compared to the general population without known genetic predisposition. The lifestyle factors, including cigarette smoking, alcohol use, obesity, and a high-fat/high-sugar diet, may contribute to late-stage diagnosis of PC by enhancing tumor progression or delayed recognition [30,31,32]. Patients with chronic pancreatitis may present with similar symptoms and difficult-to-differentiate imaging findings, leading to misdiagnosis or delayed diagnosis of PC [33]. New-onset diabetes mellitus, especially in adults over 50, can be a warning sign of underlying pancreatic cancer, potentially leading to earlier detection of the disease [34]. Other risk factors, including poor oral hygiene [35], non-alcoholic fatty liver disease [36], viral hepatitis B and C infection [37], pancreatic steatosis [38], and depression [39], are potentially modifiable or manageable, and how they are associated with the stage of PC at diagnosis remains to be determined.

While the genetic mutations in those predisposed individuals are not modifiable, some of the medical conditions can be controlled, and lifestyle factors can be modified, though this can be challenging in practice. However, identifying individuals with known risk factors for PC provides an opportunity for targeted screening in this population, thereby enhancing the accuracy and efficiency of early detection of this disease.

## 7. Social Determinants of Health and Stage of Pancreatic Cancer at Diagnosis

Socioeconomic factors such as educational level, income, health insurance, and residential location have been shown to influence the stage of PC at diagnosis (Table 2). Patients with a high education level, high income, insurance coverage, and non-rural residence are less likely to be diagnosed with PC at stage IV than those with a low educational level, low income, lack of or limited insurance coverage, and rural residence. Hypothetically, patients with low education level, low income, lack of insurance coverage, and rural location have fewer resources and access to medical care, and thus they have increased odds of being diagnosed with late-stage PC. Understanding how these social determinants impact the stage of PC at diagnosis will help inform health policy changes and facilitate early diagnosis and therapeutic interventions, thus improving patient outcomes [21].

These studies indicated the important associations among the social determinants and stage of PC at diagnosis, though with some limitations. The study by Gallegos et al. [18] is designed to identify the associations between socioeconomic factors and the stage of pancreatic cancer at diagnosis based on 256,822 patients from the National Cancer Database. The major limitation of this study is that the National Cancer Database is not a true population-based analysis. The study by Fabregas et al. [21] aims to identify the role of social determinants of health in the late diagnosis of patients with metastatic disease using univariate, multivariate logistic regression models for the evaluation of risk of a late diagnosis based on 230,877 patients from the National Cancer Database. The major limitation of this study is the risk of misclassification bias using the dataset from the National Cancer Database. The study by Segel et al. [40] is designed to estimate differences in stage of pancreatic cancer at diagnosis by rurality of patients’ residence and residence in a medically underserved area, using multivariate linear probability models of local and locoregional stage at diagnosis. The major limitation is that this study is limited to a single state, and relatively few individuals live in completely rural areas. The study by Nash et al. [41] aims to assess geographic and racial disparities in gastrointestinal cancer in a geographically and racially diverse US population using multivariate Cox proportional hazards models based on 5455 patients with pancreatic cancer in Georgia. The major limitation of this study is the lack of information on individual-level socioeconomic characteristics.

However, these social determinants can become targets for health policy intervention including (i) insurance and coverage policy by expansion of Medicaid, mandatory coverage for pancreas-protocol CT/MRI scans for those with high-risk symptoms or factors, endoscopic ultrasonography without restrictive prior authorization; (ii) providing value-based payment incentives for early detection of cancer; (iii) reimbursement for oncology navigation services, community health workers for scheduling appointment, coordination of transportation, and education about symptoms; (iv) providing mobile imaging units in underserved regions, regional clinics for rapid evaluation of pancreas, reimbursement of telehealth for consultation with gastroenterology and oncology; (v) campaigns for awareness of culturally tailored symptoms, language-specific education materials, and partnerships with community clinics, faith organization, and pharmacies.

## 8. Interactions and Mediation of Factors Associated with Stage at Diagnosis

The factors associated with the stage at diagnosis of PC form a multi-level, interacting system in which tumor biology, patient-level factors, and social determinants of health influence one another through identifiable mediating pathways. Understanding how those factors interact with one another and how they are mediated is important for identifying the barriers and reducing health disparities in cancer screening.

Emerging evidence suggests that social determinants of health contribute to the racial disparities in the stage of PC at diagnosis. An epidemiological study using data from SEER that included Black and White patients diagnosed with PC between 2005 and 2015 was conducted to examine disparities in stage at diagnosis by racial segregation [42]. Results of this study demonstrated that Black patients were more frequently diagnosed with stage IV disease than White patients. Those Black patients tended to live in more segregated areas, have higher levels of poverty, lower income, less access to insurance, and a higher rate of unemployment. Importantly, when Black patients were compared to White patients with similar levels of those socioeconomic factors, there was no significant difference in the risk of advanced stage of PC at diagnosis. This study suggested that socioeconomic factors are important mediators of the racial disparities in the stage of PC at diagnosis.

In addition, social determinants of health also contribute to delays in germline genetic testing and, thus, in the diagnosis of PC at advanced stages. Currently, for patients diagnosed with pancreatic adenocarcinoma, germline genetic testing is recommended not only for identifying genetic predisposition to malignant diseases but also for guiding the selection of systemic chemotherapy. Screening for PC is not performed on a routine basis due to its relatively low prevalence in the general population. However, those who have undergone screening have been mostly non-Latino White individuals, as reviewed [43,44]. A study using data from patients diagnosed with PC in a New York healthcare system between 2016 and 2022 was conducted to identify associations between demographic/socioeconomic factors and delays in germline genetic testing [45]. These data indicated that lower income, racial minority (African American and Hispanic), lack of supplemental health insurance, and higher needs for social work (transportation, home care) are associated with significant delays in germline genetic testing for PC. This will likely lead to delayed diagnosis at advanced stages.

**Figure 2:**
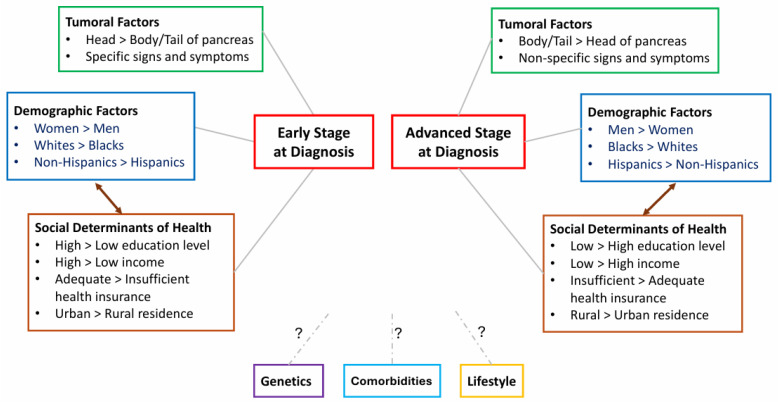
Factors associated with the stage of pancreatic cancer at diagnosis. Germline genetics, comorbidities, and lifestyle are known risk factors of pancreatic cancer, but their associations with the stage of pancreatic cancer at diagnosis are unclear.

Results of these studies suggest that socioeconomic factors are important mediators of the racial disparity in stage of PC at diagnosis, as well as germline genetic testing, which, in turn, influences stage at diagnosis (Figure 2). These findings can have practical implications, suggesting that regulatory policies of society and the healthcare system can have important impacts on the stage of PC at diagnosis, healthcare equity, and efficient use of screening resources. Further investigation that focuses on how social determinants of health interact with other factors associated with the stage of PC at diagnosis is indicated.

## 9. Screening Programs for Pancreatic Cancer

While diagnosis of PC at an early stage is crucial for curable treatment, there has been no effective screening tool or program with proven benefit for its early detection. Recently, multiple initiatives have been developed for screening and early detection of PC. The goal is to develop screening programs that target populations at risk of developing PC or even mass screening of asymptomatic populations with average risk. These include the Pancreatic Cancer Early Detection (PRECEDE) Consortium [46], PANDA (pancreatic cancer detection with artificial intelligence) [47], UK Early Detection Initiative for Pancreatic Cancer (UK-EDI) [48], EUROPAC (European Registry of Familial Pancreatic Cancer and Hereditary Pancreatitis) [49], and METAPAC [4].

These strategies are based on the recruitment of individuals at risk of developing PC with analysis of medical history, radiographical images, and biospecimens, as well as machine learning algorithms. The goals and methods of these screening initiatives are summarized in Table 3.

All of these major established programs (PRECEDE, EUROPAC, UK-EDI) focus on identifying individuals at higher-than-average risk because general population screening for PC is not currently recommended. METAPAC and PANDA focus on metabolic signatures and artificial intelligence-based imaging models, since no single definitive test for early detection exists yet. While EUROPAC and PRECEDE are designed to actively integrate clinical surveillance pathways while advancing research, METAPAC and PANDA are proof-of-concept studies that may eventually inform structured screening programs.

## 10. Early Detection Tests for Pancreatic Cancer

Complementary to those screening programs for PC, various non-invasive blood-based tests have been developed and evaluated for early detection. These tests are based on analysis of extracellular vesicles (EVs), methylation of cell-free DNA (cfDNA), protein biomarkers, patterns of cfDNA fragmentation, and protease activity. Most of these are investigational or early-stage tests; some have been validated, and many are not yet validated or FDA-approved for routine clinical screening.

Plasma-based extracellular vesicles (EVs) have been investigated as a non-invasive tool for early detection and diagnosis of PC. EVs are extracellular membrane-enclosed vesicles found in various bodily fluids. Their size ranges from 10 to 1000 nm in diameter, and they enclose cytosolic proteins, DNA, RNA, and microRNA (miRNA) as reviewed [51]. The various molecules carried by EVs have been exploited as diagnostic biomarkers for PC with excellent accuracy. Several techniques have been developed for isolation and enrichment of EVs, as reviewed [52]. Several studies have demonstrated the potential value of EV-based biomarkers for early detection of PC, as reviewed [52,53].

Notably, a large, multi-center, multinational prospective study evaluated the performance of cf-miRNA biomarkers and exosome-based miRNA for the detection of PC [54]. In this study, a training cohort of 150 patients with PC and 102 non-disease controls was analyzed in Japan. For the detection of PC, miRNA signatures in cell-free specimens and exosomes were associated with a sensitivity of 94% and 92%, respectively, and a specificity of 83% and 97%, respectively. A combination of cf-miRNA and exosomal miRNA signatures produced a sensitivity of 95% and specificity of 97% for the detection of PC. The combination of cf- and exosomal miRNA signatures for the detection of PC was validated in three independent cohorts in the United States, China, and Korea. In the United States cohort consisting of 66 patients with stage I/II PC, 62 patients with stage III/IV PC, and 193 individuals as non-disease control, the combined miRNA signatures produced an accuracy of detecting stage I/II and stage III/IV PC with an area under the curve (AUC) of 0.91 and 0.93, respectively. Furthermore, a combination of miRNA signature and CA 19-9 for the detection of PC at all stages yielded an AUC of 0.97, sensitivity of 0.95, and specificity of 0.96; for stage I/II PC, an AUC of 0.97, sensitivity of 0.91, and specificity of 0.96.

In recent years, multi-cancer early detection (MCED) tests for various malignancies, including PC, have been developed, and they are mostly based on cancer-associated molecules in blood specimens. Those molecular alterations may include methylation patterns of circulating cell-free DNA (cfDNA), circulating cell-free tumor DNA (ctDNA), tumor protein biomarkers, cancer genomic features, protease activity, and molecules within extracellular vesicles.

Galleri^®^ (GRAIL, Menlo Park, CA, USA) is a blood test that detects cancer signals by analyzing cancer-specific DNA methylation patterns in circulating cfDNA in plasma and can predict the origin of cancer signals [55,56]. It demonstrates high sensitivity for PC at advanced stages and excellent specificity. It also shows 88% accuracy for the prediction of tissue of origin to guide follow-up diagnostic steps. The Galleri^®^ test has received an FDA Breakthrough Device Designation, but it is not currently FDA-approved. It is available for healthcare providers to order, and the list price for the Galleri^®^ test is USD 949.

CancerSEEK is a multi-analyte blood test that evaluates circulating ctDNA using multiplex PCR analysis and the levels of eight cancer-associated protein biomarkers using immunoassays [57]. It aims to detect cancer at an early stage and identify the location of the cancer. CancerSEEK shows reasonable sensitivity for PC at early stages and high specificity. CancerSEEK has received a Breakthrough Device designation from the FDA for the detection of genetic mutations and proteins associated with pancreatic and ovarian cancers. The cost of the CancerSEEK test is estimated to be less than USD 500. CancerSEEK is still under development and under clinical investigation.

OncoSeek^®^ (OncoInv, Houten, The Netherlands) uses a machine learning algorithm to quantitatively analyze seven protein tumor markers in a blood specimen, along with clinical information (sex, age), for early detection of multiple cancers, including PC, as well as prediction of the possible tissue of tumor origin (70% for pancreas) [58]. Using artificial intelligence to empower OncoSeek^®^, the specificity of cancer detection was increased from 54.0% to 93.0%, and the overall sensitivity was 51.7%, resulting in an accuracy of 84.6%. This performance of OncoSeek^®^ was consistent in the training and the five validation cohorts from Brazil, China, and the United States. For PC, the sensitivity was 77.6% [59]. OncoSeek^®^ features an affordable cost of less than USD 25.

SeekInCare is a blood test that integrates the seven protein tumor markers (as used in OncoSeek) and four cancer genomic features from cfDNA by shallow whole-genome sequencing [60]. A two-step approach using OncoSeek^®^ as the initial screening test and then using SeekInCare for individuals who test positive with OncoSeek^®^. By combining OncoSeek^®^ and SeekInCare, this screening approach is able to detect PC with further increased sensitivity and specificity. The estimated cost per individual screened is USD 143. By reducing the false-positive rate and screening costs, the investigators propose this two-step approach as a cost-effective strategy for population-wide screening of cancer.

PAC-MANN-1 is a high-throughput protease-activated nanosensor assay for early detection of PC [61]. This assay distinguished PC from healthy controls and other non-cancerous pancreatic diseases by using a fluorescence-labeled protease-sensitive peptide linked with a magnetic nanosensor in order to detect protease activity in blood specimens. It is reported to detect stage I PC with high sensitivity and specificity. PAC-MANN-1 is an emerging, low-cost, rapid test with good accuracy in PC at the early stage, and it warrants further clinical development.

These MCED tests have shown promising results in the detection of PC with variably high sensitivity and specificity. A number of ongoing clinical trials and cohort studies are ongoing, and they focus on the detection of multiple cancers. Those relevant to PC include the National Cancer Institute (NCI) Early Detection Initiative for Pancreatic Cancer, NCI Vanguard Study, Pancreatic Cancer Early Detection (PRECEDE) Consortium, and ExoVita™ Pancreas Assay.

Some of these tests have been validated in large-scale prospective studies. Active clinical trials and cohort studies are ongoing to determine their clinical utility. The methodologies, sensitivities, and specificities for the detection of pancreatic cancer of those MCED tests (where available) are compared (Table 4).

## 11. Machine Learning for Screening and Early Detection

The power of early detection tests for PC can be enhanced with the application of machine learning through analysis of large medical datasets and digitized images, as reviewed [62]. Machine learning (ML) utilizes computational and statistical methods to discover patterns within datasets, including medical records, radiomics, pathomics, and molecular omics. The ability of machine learning to enhance risk prediction, detection, and diagnosis has been demonstrated across various malignancies, including PC.

Several machine learning-based models have been developed by leveraging electronic health records (EHRs) for risk prediction of PC and for identifying health conditions associated with an increased risk of PC [62,63,64,65,66,67,68,69,70,71,72] (reviewed in [68,69]). For instance, using an artificial neural network (ANN) that incorporates distinct features in health data, the ANN model is able to predict the risk of PC with a sensitivity of 87.3%, specificity of 80.8%, and an area under the receiver operating characteristic curve (AUROC) of 0.86 within the training cohort and similar results were observed within the testing cohort [63]. Through the application of ML techniques, a predictive model demonstrated the ability to identify more than 50% of patients with late-stage PC about 2 years prior to their formal diagnosis [65]. A linear regression model was established to identify individuals at risk of developing PC up to 12 months prior to a formal diagnosis, and the model demonstrated an AUC of 0.68, which was further enhanced by a feature selection strategy in EHRs [66]. In the study, a DL algorithm was used to predict the risk of developing PC, and time-sequence models using sequential neural networks, such as gated recurrence unit models and the Transformer model, were trained with disease codes extracted from EHRs. The model was able to predict the occurrence of PC within a three-year period, with an AUROC of 0.88 and 0.71 in the Danish population and the United States population, respectively [67].

To enhance the power of developing ML-based models to predict disease risk, a novel approach has been recently introduced to address imbalanced covariate shift that impacts collaborative fairness in federated learning for collaborative training of a global model without sharing raw local data [72]. This approach, FedAKD (Federated Asynchronous Knowledge Distillation), mitigates the effects of imbalanced covariate shift by excluding incorrectly predicted samples from the global model and includes clients to receive proportional gains based on their impact on model performance. Using both public datasets and a real-world EHR dataset in PC, the FedAKD was demonstrated to significantly improve collaborative fairness, enhance the accuracy of prediction of developing PC, and enhance the participation of clients. Future investigation is indicated to optimize and implement the FedAKD in combination with other screening approaches for risk prediction and early detection of PC.

Application of deep learning (DL) to the conventionally used diagnostic tools in PC, including computed tomography (CT), has been demonstrated to improve the detection and diagnosis of PC, as reviewed [62,73] as well as premalignant pancreatic cystic neoplasms [74,75,76,77,78] (reviewed in [74]) with high accuracy. For instance, a segmentation convoluted neural network (CNN) is used along with a classifier that ensembles five other CNNs for the analysis of contrast-enhanced CT images. This DL- based model can detect PC tumors <2 cm, with a sensitivity of 89.9% and a specificity of 95.9% [79,80].

The diagnostic accuracy of PC can be enhanced by leveraging DL algorithms to analyze and interpret digitized images of histopathology. For instance, a model based on a multilayer perception neural network (MNN) shows the capacity of distinguishing between malignant and benign pancreatic cytology with an accuracy of 100% [81].

These advanced computational techniques are complementary to the other screening tools for PC and are expected to facilitate screening, early detection, and diagnosis.

## 12. Discussion and Future Perspectives

**Figure 3:**
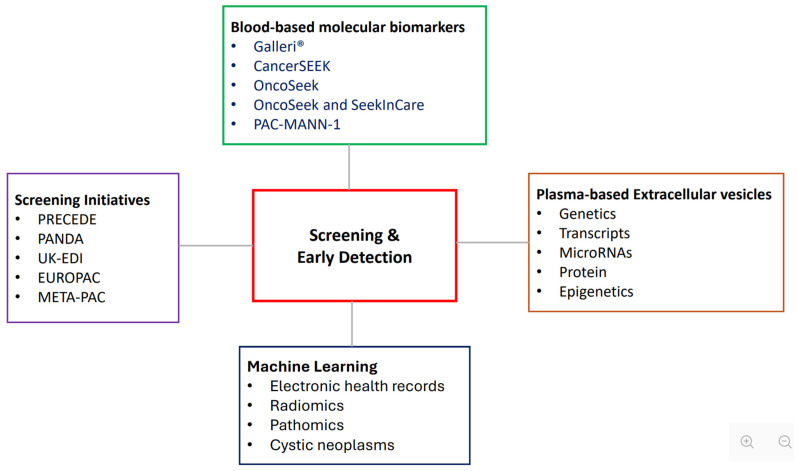
Screening strategies and tests for early detection of pancreatic cancer.

PC remains a challenging and lethal malignancy due to an asymptomatic early course, non-specific presenting symptoms, diagnosis mostly at advanced stages, systemic treatment with limited efficacy, and lack of a widely adopted and effective screening test and program. This review highlights the factors associated with the stage of PC at diagnosis, including tumoral factors, demographic factors, social determinants of health, and how they interactively influence stage at diagnosis (Figure 2). The various screening initiatives for PC were examined, the emerging biomarker-based blood tests for early detection compared, and advances in the application of machine learning were described (Figure 3).

Though tumor-related signs and symptoms are relatively non-specific, expert-guided education and awareness among the general population about the clinical presentations of PC may help improve its recognition and increase the likelihood of its early diagnosis. For individuals with hereditary genetic abnormalities as well as risky lifestyle factors and comorbidities, their link with the stage of PC at diagnosis remains to be investigated. However, understanding the association of these factors with the stage of PC at diagnosis will make a positive impact on the efficiency of screening strategies for early detection of the disease.

Epidemiological studies have demonstrated that certain factors tend to be associated with the diagnosis of PC at the advanced stage, including Blacks, men, low income, low education level, insufficient insurance, and rural residence. Those social determinants of health are also important mediators of racial disparity in stage at diagnosis and germline genetic testing. Understanding these associated factors and mediators may help develop policy interventions, risk-stratified eligibility, and electronic health records-based triggering for screening of PC, as well as tailoring validation of biomarker testing in high-risk groups most likely to present late in the disease course.

The advent of the emerging EV-based and MCED tests offers an important opportunity for early detection and diagnosis of PC. While these tests show promising sensitivity and specificity, especially in advanced disease, their clinical implementation faces key limitations: cost, accessibility, predictive value in asymptomatic populations, and the risks of overdiagnosis and psychological burden. Moreover, no randomized controlled trial to date has demonstrated reductions in PC-specific mortality with these tests, highlighting the gap between technological promise and real-world impact. Specifically for PC, EV-based, and MCED testing may be most useful in high-risk populations (e.g., familial syndromes, new-onset diabetes in elderly adults), where predictive value is enhanced, and unnecessary harm may be reduced. This further underscores the importance of identifying and understanding the factors associated with the stage of diagnosis as reviewed and discussed in this article.

Recently, prediction models based on nullomers have emerged as unique and powerful tools for early detection of multiple types of cancer [82]. Nullomers are short sequences that are absent from the human genome, and they can emerge as a result of somatic mutations in cancer. Nullomers can be DNA, RNA, or amino acid sequences [83,84]. Cell-free nullomers have been identified from over 10,000 matched tumor-normal blood specimens. Preliminary evidence indicates that nullomers in cell-free RNA enabled the detection of multiple types of tumors, and multiple tumor classification models with an AUC >0.9, including a classifier for hepatocellular carcinoma with an AUC >0.99 [84]. An ongoing investigation using a combination of nullomers in cell-free DNA, RNA, and peptides is ongoing by utilizing blood specimens from patients with PC and individuals without PC as controls.

Machine learning-based algorithms are powerful tools that not only enhance the ability to predict the risk of developing PC but also improve test sensitivity and specificity for early detection of PC. However, the datasets used in machine learning studies of PC present challenges that can limit performance, reduce generalizability, and introduce algorithmic bias. These dataset challenges may include limited size and diversity, class imbalance, inconsistency of annotation, and lack of transparency and standardization. These can lead to over-estimation or under-estimation of the risk of PC in sub-groups, under-performance of early detection of PC in the real world, and persistent health disparities when models are applied across varied clinical settings. These challenges may be addressed through multi-institutional collaborations and federated learning to pool data from diverse populations; standardized protocols for annotating diagnoses and disease stages in PC; evaluation of model performance across demographic subgroups of patients; and transparent documentation of datasets and models.

Conceivably, mass screening for PC in asymptomatic and both average- and high-risk populations can be accomplished by combining multiple modalities. These include (i) education of the general public about biology and clinical presentations of PC, (ii) deeper understanding of the factors associated with stage at diagnosis, (iii) improved development and validation of early detection tests and screening programs, (iv) detection of pancreatic pre-malignant lesions, (v) machine learning-based algorithms using electronic health records for risk assessment, (vi) developing non-invasive and accurate imaging tests in combination with radiomic analysis, and (vii) application of multimodal machine learning.

A new framework for screening and early detection of PC will consist of machine learning-driven risk modeling by integrating social determinants of health, clinical, and genomic data; study design by enriching trials with high-risk individuals and evaluating biomarker-guided screening strategies; and health system interventions with automated alerts for high-risk phenotypes and reduced diagnostic delays. We propose research to test the central hypothesis that integrating multi-level risk factors with assays for early detection will enable the identification of high-risk individuals and improve the stage of PC at diagnosis. This proposed research will be innovative by multimodal risk modeling that integrates longitudinal EHR data, germline and somatic genomics, imaging-derived features, and social determinants of health (education, income, geography, insurance); stage-shift prediction by determining the proportion of early-stage vs. late-stage detection/diagnosis, and time-to-diagnosis trajectories; machine learning-guided deployment of tests for early detection of PC using blood-based assays and imaging; addressing systemic disparities in stage of PC at diagnosis with incorporation of social determinants of health, detection bias, and evaluation across racial and socioeconomic strata.

However, early detection strategies for PC must not only be effective and efficient but also socially equitable. Without deliberate policy and practice interventions, emerging technologies may widen disparities by being available only to affluent or well-insured groups. Efforts to integrate EV-based and MCED testing into community-based programs, mobile clinics, and safety-net systems are critical to ensure that innovations improve outcomes for the populations most burdened by this disease.

## 13. Conclusions

The stage of PC at diagnosis critically impacts treatment options and, consequently, clinical outcomes. Currently, there is no established screening program for its early detection. Tumoral and demographic factors, as well as social determinants of health, are associated with the stages of PC at diagnosis. Whites, high education levels, and income, as well as adequate health insurance, tend to be associated with early-stage diagnosis. Men, blacks, low education levels and income, and residing in rural or underserved areas tend to be associated with advanced-stage diagnosis. Socioeconomic factors are important mediators of racial disparities in stage at diagnosis as well as germline genetic testing. Screening initiatives and blood-based molecular biomarker tests for early detection of pancreatic cancer, as well as machine learning-based models for risk prediction and imaging diagnostics, are emerging. By determining and understanding the factors associated with the stage at diagnosis, risk-stratified screening can be developed by combining demographics, genetics, comorbidities, lifestyle, and social determinants. Regulatory policies that address the social determinants of health can guide the development of screening strategies to allocate resources for equitable access to healthcare and to reduce disparities in patients with PC.

## Figures and Tables

**Table 1:** Stage of pancreatic cancer at diagnosis by race and ethnicity. The exact stages are not available in the cited literature. For practical purposes, pancreatic cancer is classified as localized/loco-regional/early (resectable with curative intent) vs. distant/advanced/late stages (unresectable or metastatic).

Studies	Stage of Pancreatic Cancer at Diagnosis
Saif et al., 2005 [16]	Design: To examine distributions across stages of pancreatic cancer between Blacks and Whites based on 530 eligible patients from the institutional database of the authors. Finding: No significant difference in the distribution of tumor stage between Whites and Blacks. Key Limitation: Study using the database of a single institution
Noel and Fiscella, 2019 [19]	Design: A narrative review of health disparities in pancreatic cancer based on 1,658,370 new cancer cases from SEER data Finding: Whites: 37% loco-regional disease, 52% distant disease. Blacks: 34% loco-regional disease, 57% distant disease. Key Limitation: Retrospective study with narrative review of literature
Blanco et al., 2021 [20]	Design: To examine the impact of racial segregation on the diagnosis of pancreatic cancer using the racial index of dissimilarity based on 60,172 black and white patients in urban counties identified using data from the SEER Program and the 2010 Census. Finding: Blacks with more advanced-stage disease (63.5% vs. 58.3%; *p* < 0.05). Key Limitation: Retrospective study with static county-level estimates
Fabregas et al., 2022 [21]	Design: To identify the role of social determinants of health in late diagnosis of patients with metastatic disease using univariate, multivariate logistic regression models for evaluation of risk of a late diagnosis based on 230,877 patients from the National Cancer Database. Finding: Blacks have a 9% higher frequency of advanced disease than Whites. Key Limitation: Risk of misclassification bias using the dataset from the National Cancer Database.
Gallegos et al., 2023 [18]	Design: To identify the associations between socioeconomic factors and the stage of pancreatic cancer at diagnosis based on 256,822 patients from the National Cancer Database Finding: Blacks have a 10% higher likelihood of advanced stage disease than Whites (odds ratio 1.106; 95% CI = 1.069–1.144; *p* < 0.001). Hispanics have 5% higher odds of stage IV disease compared to non-Hispanics (odds ratio = 1.052, 95% CI = 1.003–1.104, *p* < 0.039). Other races have 15% higher odds of an early-stage disease compared to Whites (odds ratio = 0.849, 95% CI = 0.804–0.897, *p* < 0.001). Key Limitation: The National Cancer Database is not truly a population-based analysis.
Fagenson et al., 2020 [22]	Design: To analyze whether racial and ethnic disparities in pancreatic cancer exist using Cox proportional hazard regression models based on 36,756 patients in the Florida Cancer Data System Finding: Blacks are diagnosed at a younger age and at a later stage, with worse survival outcomes than Whites. Key Limitation: Key confounders not included in the data from the Florida Cancer Data System and a lack of control in selecting and reporting those key variables.
Schiefelbein et al., 2022 [23]	Design: To investigate race and ethnicity-based disparities in patients with pancreatic cancer in Wisconsin using an adjusted logistic regression model based on 8490 patients in the Wisconsin Cancer Reporting System. Finding: Non-Hispanic Blacks: 12.4% localized disease. Non-Hispanic Whites: 9.6% localized disease. Similar proportion of non-Hispanic Blacks and non-Hispanic Whites with distant disease (53.1%). Key Limitation: Potential biological and social confounding factors available.

**Table 2:** Stage of pancreatic cancer at diagnosis by socioeconomic factors.

Socioeconomic Factors	Stage of Pancreatic Cancer at Diagnosis
Educational Level	Patients with >93% completion of high school are less likely to have a late-stage diagnosis than those with under 82.4% completion [21]. Patients with lower levels of education (no high school diploma) had increased rates of stage IV diagnoses, whereas those with higher levels of education had early-stage disease (*p* < 0.002) [18].
Income	Average incomes <USD 40,277 had higher odds of stage IV disease as compared to those from areas with incomes >USD 63,333 [21]. Median household income below USD 38,000 and in postal codes with lower high school graduation rates had higher rates of late-stage diagnoses (*p* < 0.025) [18].
Health Insurance	A stage IV diagnosis was significantly higher for those without insurance (odds ratio 1.52) compared to those with Medicare or private insurance [21]. Patients with private or Medicaid insurance had higher rates of late-stage diagnoses than those with other types of insurance [18]. All other insurance types showed lower rates of late-stage diagnosis compared to uninsured patients (*p* < 0.001) [18,21].
Residential Location	Residents in medically underserved and rural areas in Pennsylvania were more likely to present with advanced-stage disease, with no case of localized stages reported in completely rural areas [40] In Georgia, patients residing in urban counties were more likely to be diagnosed with localized disease than those in rural areas [41] Patients residing in rural areas were more likely to be diagnosed with stage IV disease as compared to those in urban areas [21]

**Table 3:** Screening initiatives for advancing early detection of pancreatic cancer. These initiatives have certain overlaps, and they are developed and conducted at various institutions across different countries. This inclusion of people from different races and ethnicities may enable future application of the findings to broad populations.

Initiatives	Goals	Design and Methods	Reference
PRECEDE	Enable surveillance for PC tailored to risk category	An observational prospective cohort study by a multi-institutional international collaboration with 1400 participants enrolled in the highest-risk cohort through longitudinal standardized clinical data, imaging, and germline testing	[46]
PANDA	Develop non-contrast CT as a large-scale screening tool for PC	A deep learning approach is trained on a dataset of 3208 patients from a single center to detect and classify pancreatic lesions using non-contrast CT scans and validated across 10 centers involving 6239 patients	[47]
UK-EDI	Develop molecular, epidemiological, and demographic biomarkers for early detection of PC in high-risk new-onset diabetes (NOD group	A national, prospective, observational cohort study that aims to recruit 2500 individuals with new-onset diabetes (<6 months postdiagnosis) through clinical information and biospecimen	[48]
EUROPAC	Develop a tailored screening pathway for individuals with a germline genetic cause of PC	An international prospective observational study to recruit and screen individuals (10,000 individuals in the United Kingdom) with either familial PC or hereditary pancreatitis Risk stratification by age, diabetes, smoking, and alcohol; screening by endoscopic ultrasonography, CT, and MRI scans	[49]
METAPAC	Develop a surveillance tool for patients with a low risk of developing PC to avoid unnecessary invasive procedures and enable early detection of PC	A prospective, multicenter, investigator-masked, enrichment design study across 23 centers in Germany involving 1129 patients Patients with CT-identified pancreatic lesions that required further diagnostic assessment were recruited and analyzed using targeted quantitative plasma metabolites.	[50]

**Table 4:** Comparison of the methodology, sensitivity, and specificity of multi-cancer early detection (MCED) tests for pancreatic cancer.

Test	Design and Methodology	Sensitivity	Specificity	Key Limitation	Reference
Galleri^®^	A case–control study with 4077 participants in an independent validation set using an MCED test for methylation patterns of cfDNA.	83.7% (overall) 61.9% (stage I) 60.0% (stage II) 85.7% (stage III) 95.9% (stage IV)	99.0%	The possibility of increased tumor cfDNA fraction in blood samples collected following biopsy of cancer relative to pre-biopsy.	[55,56]
CancerSEEK	A case–control study involving 1005 patients using multiplex PCR analysis of ctDNA mutations and protein biomarkers.	70% (stages I–III)	>99%	The participants had mostly been diagnosed with symptomatic cancer, such that the sensitivity of detection is likely lower in asymptomatic individuals with earlier-stage disease.	[57]
OncoSeek^®^	An observational study involving 7565 participants using a machine learning algorithm to retrospectively analyze a panel of seven selected protein tumor markers consisting of AFP, CA 125, CA 15-3, CA 19-9, CA 72-4, CEA, and CYFRA 21-1 in a blood-based MCED test.	77.6% (all stages)	90%	The participants had mostly been diagnosed with symptomatic cancer, such that the sensitivity of detection is likely lower in asymptomatic individuals with probably earlier-stage disease.	[58]
OncoSeek^®^ and SeekInCare	A case–control study involving a total of 1197 participants using a two-step MCED approach, with OncoSeek initially used for primary screening, and then SeekInCare used for testing on the positive results from OncoSeek^®^ to further evaluate the cancer risk by integrating OncoSeek^®^ results with cancer-specific genomic features from cfDNA.	82.4% (case–control) 92.3% (real-world cohort)	97.7%	Simulations drawn from retrospective data and a simulated MCED screening cohort, rather than real-world data, were used in this study.	[60]
PAC-MANN-1	A longitudinal cohort of patients undergoing surgical removal of the primary tumor and a separate blinded retrospective study using a magnetic nanosensor assay to measure serum cancer-associated protease cleavage of a target-probe nanosensor with a fluorescent readout.	85% (stage I)	96%	The cost of the test for population-based screening	[61]

## Data Availability

No new data were created or analyzed in this study. Data sharing is not applicable to this article.

## Abbreviations

The following abbreviations are used in this manuscript:

PC	Pancreatic cancer
SEER	Surveillance, Epidemiology, and End Results
EOPC	Early-onset pancreatic cancer
PRECEDE	Pancreatic Cancer Early Detection
PANDA	Pancreatic cancer detection with artificial intelligence
UK-EDI	UK Early Detection Initiative for Pancreatic Cancer
EUROPAC	European Registry of Familial Pancreatic Cancer and Hereditary Pancreatitis
MCED	Multi-cancer early detection
cfDNA	Cell-free DNA
ctDNA	Circulating tumor DNA
